# A Single-Center Retrospective Study to Identify Causes of Sex Differences in the Living Kidney Donor Evaluation Process

**DOI:** 10.34067/KID.0000000581

**Published:** 2024-09-16

**Authors:** Ritah R. Chumdermpadetsuk, Adriana Montalvan, Stalin Canizares, Bhavna Chopra, Martha Pavlakis, David D. Lee, Devin E. Eckhoff

**Affiliations:** 1 Beth Israel Deaconess Medical Center Department of Surgery, Boston, MA; 2 Beth Israel Deaconess Medical Center Department of Medicine, Boston, MA

**Keywords:** gender difference, kidney donation, kidney transplantation, obesity, renal transplantation, transplantation

## Abstract

**Key Points:**

- Female overrepresentation in living kidney donation stems from higher self-referral rates, not differences in approval or follow-through.
- Male volunteers are not more likely to be declined as donors due to medical contraindications, contrary to common assumptions.
- Engaging more male volunteers in living donation could expand access to kidney transplantation and reduce waitlist times.

**Background:**

Multiple studies have shown that female volunteers are living donors (LDs) for kidney transplantation at higher rates than male volunteers. However, the underlying reasons for this observation are not well understood. We examined the LD evaluation process to determine the point at which sex imbalance arises. On the basis of a previous study, we hypothesized that both sexes are equally likely to become approved as LDs, but female volunteers are more likely to follow through with donation.

**Methods:**

This is a single-institution retrospective chart review of self-referrals for LD evaluation between January 2009 and December 2022. Self-referrals were identified using the Organ Transplant Tracking Record database and cross-referenced with billing data. Exclusion at each stage of evaluation was recorded and compared between sexes using log binomial regression; unadjusted and adjusted (for donor age, race, ethnicity, relationship to recipient, and recipient sex) risk ratios with 95% confidence interval were determined.

**Results:**

One thousand eight hundred sixty-one self-referrals were reviewed, including 1214 female (65.2%) and 647 male (34.8%) volunteers, resulting in 146 approvals and 125 donations (76/125, 60.8% female, 49/125, 39.2% male). Adjusted risk ratios indicated no significant differences between sexes in completing medical and/or psychosocial workup, having medical and/or psychosocial contraindications, being approved for donation, and proceeding with donation. The top medical contraindications for both sexes were obesity, hypertension, and nephrolithiasis.

**Conclusions:**

Female overrepresentation among LDs is likely due to the 1.9 times higher rate of self-referral for evaluation. After this point, both sexes were equally likely to complete workup, be approved, and follow through with donation. Increased efforts to engage male volunteers at the initial self-referral stage has the potential to expand access to LD kidney transplantation.

**Podcast:**

This article contains a podcast at https://dts.podtrac.com/redirect.mp3/www.asn-online.org/media/podcast/K360/2024_12_26_KID0000000581.mp3

## Visual Abstract

**Figure:**
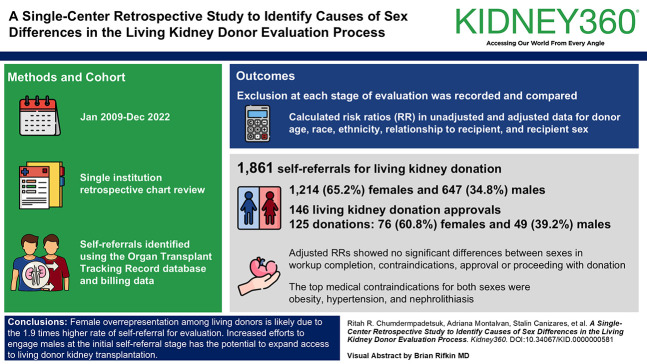

## Introduction

In 2022, there were 140,165 individuals on the kidney transplantation (KT) waitlist.^1^ Living donor KT (LDKT) is an excellent strategy to address the shortage of organs, while providing better outcomes than deceased donor KT.^2–4^ It has been established that female volunteers are living donors (LDs) at higher rates than male volunteers.^4–12^ However, the underlying reasons for this are not well understood. Several hypotheses have been put forward^6,8,9^ with little data to support them because most studies include only individuals who underwent donor nephrectomy and not those who were excluded throughout the evaluation process.^6–12^ Therefore, barriers to donation which may account for the observed sex imbalance could not be directly discerned. As a notable exception, a 2005 study at our center in Massachusetts, United States, comprising over 500 potential living donors (PLDs), found that while both sexes were equally likely to become approved, female volunteers were more likely to follow through with living donation.^4^

Identifying the causes of this phenomenon has the potential to inform the design of targeted interventions for increasing male living donation, thus expanding access to LDKT. Therefore, we sought to examine the LD evaluation process in recent years to determine the point at which the sex imbalance arises. Possible explanations included more frequent exclusion of male volunteers because of medical contraindications^6^ and/or positive cross-matches with their female spouses from HLA sensitization during pregnancy,^6^ as well as greater willingness among female volunteers to engage in the process. On the basis of the aforementioned study,^4^ we initially hypothesized that both sexes are equally likely to become approved as LDs, but female volunteers are more likely to follow through.

## Methods

### Participant Selection

Adults volunteering for LD evaluation for KT adult candidates listed at our institution between January 2009 and December 2022 were included; pediatric transplants are not performed at this center. PLDs were identified using the Organ Transplant Tracking Record database, cross-referenced with billing data queried for organ donation–related diagnoses and procedures (International Classification of Diseases, Tenth Revision Codes Z00.5 and Z52.4,^13^ Current Orocedural Terminology code 50320). PLDs for nondirected (*n*=124) and paired (*n*=37) donations were excluded. PLDs were excluded where the intended recipient could not be found in the medical record (*n*=59) and/or the sex of the PLD was unavailable (*n*=42). This resulted in a cohort of 1861 PLDs.

Of the 124 excluded nondirected PLDs (83 female [66.9%], 41 male [33.1%]), two resulted in donation (one female, one male). The low conversion rate (1.6%) suggested substantial differences in their motivations, commitment, and circumstances compared with directed LDs. Consequently, we chose to exclude them from our cohort.

Of the 37 excluded PLDs for paired exchange (27 female [73.0%], 10 male [27.0%]), 12 resulted in donation (seven female, five male). Because the matching process often involves waiting, most approved individuals likely have not had sufficient time to donate. This delay is beyond their control, making it difficult to assess whether any lack of follow-through is intentional. Evaluated individuals who were declined because of ABO incompatibility (ABOi) and/or a positive cross-match with their intended recipient, and did not subsequently enter the paired exchange program, were not excluded.

### Living Kidney Donor Referral Process

KT candidates are provided a link to our institutional LD online intake form, which they disseminate to PLDs. The form serves as the initial self-referral for evaluation and includes the name, date of birth, age, contact information, and comorbidities of PLDs. In addition, it informs PLDs that they would be ineligible if they had diabetes mellitus, HIV, or were taking >2 medications for hypertension. We lack the mechanism for capturing individuals who began but did not complete the intake form, whether due to realizing that they would be ineligible or other reasons. Thus, these individuals were not included in the study.

Once submitted, the form is reviewed by a nurse coordinator who contacts PLDs and conducts more detailed screening through phone with the aid of a standardized checklist. The nurse coordinator directs PLDs to the nearest blood collection laboratory, where they are tested for ABOi. If no contraindications are found at this point, PLDs are brought into the hospital for further evaluation with a multidisciplinary team consisting of a nephrologist, transplant surgeon, and social worker.

There is no institutional protocol for situations involving multiple PLDs for the same recipient. In these scenarios, a maximum of three are assessed simultaneously. Priority is given to those with fewer comorbidities, direct matches, and who are located closer to our center. Once the first PLD is approved, all other evaluations are halted. Other than those stated on the intake form, our exclusion criteria during the study period included body mass index (BMI) >32, more than one lifetime occurrence of nephrolithiasis, and concern for malignancy.

### Study Design

Retrospective chart review of the LD evaluation process (Institutional Review Board protocol 2023P001162) was conducted by one reviewer. The demographic and socioeconomic characteristics of PLDs were recorded. Binary outcome data were collected on each step of the evaluation, serving as end points in our statistical analysis (see below). Medical and psychosocial contraindications were recorded when applicable. The clinical and research activities being reported follow the Principles of the Declaration of Istanbul as outlined in the Declaration of Istanbul on Organ Trafficking and Transplant Tourism and the Strengthening the Reporting of Observational Studies in Epidemiology guidelines.

### Statistical Analyses

PLDs were categorized into groups on the basis of sex. We examined the following end points: undergoing blood testing, being ABOi with the recipient, having a positive cross-match with the recipient, completing medical and/or psychosocial workup, having a medical and/or psychosocial contraindication, being approved for donation, and proceeding with the donation. The risk ratio (RR) for each end point comparing female with male volunteers was calculated using log binomial regression, with a 95% confidence interval. Both unadjusted and adjusted (for donor age, race, ethnicity, relationship to recipient, and recipient sex) RRs were determined. Medical and psychosocial contraindications were ranked from most to least frequent within each cohort, calculated as percentages of individuals who were excluded because of contraindications, and compared between cohorts using chi-square analysis.

The cohort was divided into groups of female donors (FDs), male donors (MDs), female non-donors (FNDs), and male non-donors (MNDs). Demographic and socioeconomic variables were compared between groups, with FD as the reference, using chi-square analysis. The age ranges used were in accordance with the Organ Procurement and Transplantation Network/Scientific Registry of Transplant Recipients Annual Data Report. More granular socioeconomic variables (educational level, employment status, expression of financial concern, and health insurance status) were compared between FD and MDs only because these were unavailable for most of the non-donors. All *P* values < 0.05 were considered statistically significant. Analysis was conducted with STATA 18 software (StataCorp).

## Results

### Living Kidney Donor Evaluation Process

In total, 1861 PLDs were reviewed, including 1214 female (65.2%) and 647 male (34.8%) volunteers; females were 1.9 times more likely to volunteer for LD evaluation. Table 1 presents the RRs for various stages of the evaluation process, comparing female with male volunteers. Both the unadjusted and adjusted RRs indicated no significant differences between sexes in undergoing blood testing and having a positive cross-match with the recipient. In addition, there were no significant differences in the risk of completing medical workup or having a medical contraindication. Although the unadjusted RR suggested that female volunteers had a lower risk of completing psychosocial workup, this difference was not observed in the adjusted RR. Upon completing the psychosocial workup, there was no difference in the risk of having a psychosocial contraindication. Male and female volunteers had no significant difference in the risk of being approved for donation and following through once approved. Furthermore, the risk of proceeding with donation from the point of entering the PLD pool (after submitting the online intake form) was not significantly different between sexes.

**Table 1:** Risk ratios for stages of living kidney donor evaluation comparing female with male volunteers

Evaluation Stage	Male (*n*=647) (%)	Female (*n*=1214) (%)	Unadjusted RR (95% CI)	Adjusted RR (95% CI)
**Blood test (%)**	225 (34.8)	382 (31.5)	1.05 (0.98 to 1.12)	0.99 (0.93 to 1.05)
ABO incompatible (%)	32 (14.2)	112 (29.3)	2.11 (1.47 to 3.03)	1.93 (1.34 to 2.77)
Positive cross-match (%)	4 (1.8)	6 (1.6)	0.88 (0.25 to 3.10)	0.57 (0.11 to 2.95)
**Completed medical workup (%)**	245 (37.9)	434 (35.8)	0.94 (0.83 to 1.07)	0.93 (0.82 to 1.06)
Medical contraindication (%)	184 (75.1)	339 (78.1)	1.05 (0.96 to 1.15)	1.03 (0.95 to 1.12)
**Completed psychosocial workup**	134 (20.7)	205 (16.9)	0.82 (0.67 to 0.99)	0.85 (0.71 to 1.03)
Psychosocial contraindication (%)	32 (23.9)	57 (27.8)	1.17 (0.81 to 1.71)	1.14 (0.80 to 1.63)
Recipient too sick, deceased, or declined gift (%)	8 (1.2)	39 (3.2)	2.60 (1.22 to 5.53)	2.51 (1.18 to 5.36)
**Approved (%)**	58 (9.0)	88 (7.3)	0.81 (0.59 to 1.11)	0.86 (0.63 to 1.17)
Donated (%)	49 (84.5)	76 (86.4)	1.02 (0.89 to 1.17)	0.98 (0.85 to 1.13)
Donated (%)	49 (7.6)	76 (6.7)	0.83 (0.58 to 1.17)	0.86 (0.61 to 1.21)

CI, confidence interval; RR, risk ratio.

Female volunteers had a higher risk of being excluded as a LD because of ABOi with their intended recipients in both the unadjusted (RR, 2.11 [1.47 to 3.03]) and adjusted (RR, 1.93 [1.34 to 2.77]) calculations and because of their recipient being too sick, deceased, or declining the gift in both the unadjusted (RR, 2.60 [1.22 to 5.53]) and adjusted (RR, 2.51 [1.18 to 5.36]) calculations.

**Figure 1:**
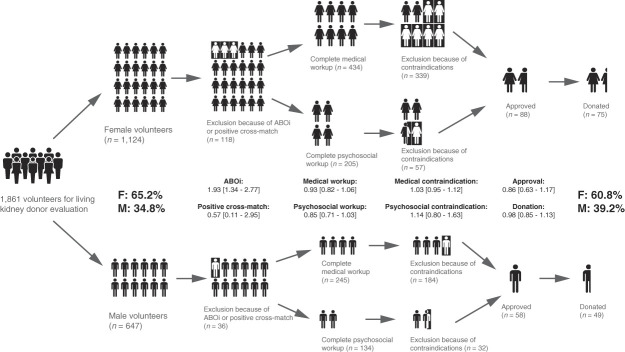
**Graphical representation of the living kidney donor evaluation process.** ABOi, ABO incompatibility; F, female; M, male.

The evaluation process is graphically depicted in Figure 1. There was no fixed order for completing the medical and psychosocial evaluations because they were typically conducted on the basis of the availability of health care providers and volunteers. Thus, Figure 1 illustrates these components as parallel pathways in the workflow diagram.

### Medical and Psychosocial Contraindications

Table 2 presents the ranking of medical contraindications, stratified by sex (*n*=339 excluded female volunteers, 184 excluded male volunteers). The most prevalent medical contraindication for both sexes was BMI >32, accounting for the exclusion of 81 male (44.0%) and 182 female (53.7%) volunteers; female volunteers were significantly more likely to be declined because of this contraindication (*P* = 0.035). The second and third contraindications were hypertension (uncontrolled on >2 medications) and more than one lifetime occurrence of nephrolithiasis. The fourth to sixth contraindications for male volunteers were cardiovascular disease excluding hypertension (for which they were significantly more likely to be declined, *P* = 0.004), concern for malignancy, and diabetes mellitus and for female volunteers were concern for malignancy, diabetes mellitus, and autoimmune disease. These accounted for the exclusion of 151 male (82.1%) and 271 female (79.9%) volunteers. Table 3 presents the ranking of psychosocial contraindications, stratified by sex (*n*=57 excluded female volunteers, 32 excluded male volunteers). The top five contraindications accounted for the exclusion of 27 of 32 male volunteers (84.4%) and 48 of 57 female volunteers (84.2%).

**Table 2:** Ranking and comparison of medical contraindications, stratified by sex

Contraindication	Rank (M)	Male (*n*=184) (%)	Rank (F)	Female (*n*=339) (%)	*P* Value
BMI >32 kg/m^2^	1	81 (44.0)	1	182 (53.7)	0.035
Hypertension^a^	2	27 (14.7)	2	33 (9.7)	0.091
Nephrolithiasis^b^	3	19 (10.3)	3	32 (9.4)	0.74
Cardiovascular disease (excluding hypertension)	4	17 (9.2)	8	11 (3.2)	0.004
Concern for malignancy	5	11 (6.0)	4	22 (6.5)	0.82
Diabetes mellitus	6	10 (5.4)	5	16 (4.7)	0.72
Clotting disorder and/or history of deep vein thrombosis	7	7 (3.8)	9	11 (3.2)	0.74
Neurological disease	8	6 (3.3)	7	12 (3.5)	0.87
Respiratory disease	9	6 (3.3)	12	7 (2.1)	0.40
Chronically on nephrotoxic medications	10	5 (2.7)	10	9 (2.7)	0.97
Renal anatomy	11	5 (2.7)	13	7 (2.1)	0.63
Concern for bloodborne infection	12	4 (2.2)	17	3 (0.9)	0.22
Autoimmune disease	13	3 (1.6)	6	13 (3.8)	0.16
Low GFR^c^	14	3 (1.6)	11	9 (2.7)	0.46
Elderly^d^	15	2 (1.1)	14	5 (1.5)	0.71
Disability	16	2 (1.1)	18	2 (0.6)	0.53
Complex abdominal surgical history	17	2 (1.1)	24	0 (0)	0.054
Chronic anemia	18	1 (0.5)	19	2 (0.6)	0.95
Gastrointestinal issue	19	1 (0.5)	20	2 (0.6)	0.95
Opioid dependence	20	1 (0.5)	25	0 (0)	0.17
Chronic pain and/or fatigue	21	0 (0)	15	4 (1.2)	0.14
Bleeding disorder	22	0 (0)	16	4 (1.2)	0.14
Family history of IgA nephropathy	23	0 (0)	21	2 (0.6)	0.30
Malnutrition	24	0 (0)	22	2 (0.6)	0.30
Proteinuria	25	0 (0)	23	1 (0.3)	0.46

BMI, body mass index; F, female; M, male.

a Uncontrolled on >2 medications.

b More than one kidney stone per lifetime.

c Adjusted for body surface area.

d While no official age-based exclusion criterion existed, those older than 75 years were not typically invited for further evaluation.

**Table 3:** Ranking and comparison of psychosocial contraindications, stratified by sex

Contraindication	Rank (M)	Male (*n*=32) (%)	Rank (F)	Female (*n*=57) (%)	*P* Value
Substance use disorder	1	11 (34.4)	2	12 (21.1)	0.17
Ambivalence	2	6 (18.8)	4	7 (12.3)	0.41
Concerns regarding young age (<21 yr)	3	5 (15.6)	1	19 (33.3)	0.071
International and/or lives far from the recipient	4	4 (12.5)	5	7 (12.3)	0.98
Depression	5	3 (9.4)	11	1 (1.8)	0.096
Anxiety	6	3 (9.4)	12	1 (1.8)	0.096
Lack of social support	7	2 (6.3)	6	5 (8.8)	0.67
Cognitive impairment	8	2 (6.3)	16	0 (0)	0.056
History of suicidal ideation and/or attempt	9	1 (3.1)	10	1 (1.8)	0.68
Financial incentive	10	1 (3.1)	15	0 (0)	0.18
Post-traumatic stress disorder	11	1 (3.1)	3	9 (15.8)	0.069
Unstable and/or difficult relationship with the recipient	12	0 (0)	8	2 (3.5)	0.28
Eating disorder	13	0 (0)	9	2 (3.5)	0.28
Bipolar disorder	14	0 (0)	7	2 (3.5)	0.28
Financially unstable	15	0 (0)	14	1 (1.8)	0.45
Unresponsive to the recipient care team	16	0 (0)	13	2 (3.5)	0.45

F, female; M, male.

### Comparison of Characteristics between Potential Living Kidney Donors

Table 4 summarizes the characteristics of PLDs, divided into groups of FDs, MDs, FNDs, and NMDs (*n*=76, 49, 1,138, and 598, respectively), with comparison between FDs and other groups. There was no significant difference in race between FDs and MDs. However, a higher percentage of FDs were White compared with FNDs (*P* = 0.040) and MNDs (*P* = 0.019). Ethnicity did not show significant differences across groups. FDs tended to be older, with 48.7% (37/76) in the 40- to 54-year age range compared with approximately 35% in other groups, although this was not statistically significant.

**Table 4:** Comparison of demographic and socioeconomic variables between potential living kidney donors

Characteristic	FD (%)	MD (%)	FND (%)	MND (%)
**Race**				
Non-White	7 (9.2)	8 (16.3)	203 (18.5)	114 (20.5)
White	69 (90.8)	41 (83.7)	892 (81.5)	443 (79.5)
Total	76 (100.0)	49 (100.0)	1095 (100.0)	557 (100.0)
*P* value	—	0.23	0.040	0.019
**Ethnicity**				
Hispanic	4 (5.3)	4 (8.2)	93 (8.5)	59 (10.5)
Non-Hispanic	72 (94.7)	45 (91.8)	1001 (91.5)	504 (89.5)
Total	76 (100.0)	49 (100.0)	1094 (100.0)	563 (100.0)
*P* value	—	0.52	0.32	0.15
**Age range, yr**				
18–29	10 (13.2)	7 (14.3)	218 (19.2)	103 (17.2)
30–39	12 (15.8)	12 (24.5)	221 (19.5)	114 (19.1)
40–54	37 (48.7)	18 (36.7)	393 (34.6)	215 (35.9)
55 or older	17 (22.4)	12 (24.5)	303 (26.7)	166 (27.8)
Total	76 (100.0)	49 (100.0)	1135 (100.0)	598 (100.0)
*P* value	—	0.53	0.096	0.20
**Relationship with the intended recipient**				
Spouse	11 (14.5)	6 (12.2)	50 (4.6)	27 (4.9)
Parent	7 (9.2)	8 (16.3)	65 (6.0)	50 (8.9)
Child	3 (4.0)	2 (4.1)	25 (2.3)	12 (2.2)
Sibling	19 (25.0)	14 (28.6)	82 (7.6)	96 (17.3)
Other, related	4 (5.3)	6 (12.2)	110 (10.2)	54 (9.7)
Other, not related	32 (42.1)	13 (26.5)	749 (69.3)	317 (57.0)
Total	76 (100.0)	49 (100.0)	1081 (100.0)	556 (100.0)
*P* value	—	0.38	<0.001	0.004

Characteristic	FD (%)	MD (%)
**Education**		
Less than high school	0 (0.0)	5 (10.6)
High school	18 (27.7)	14 (29.8)
Undergraduate	31 (47.7)	19 (40.4)
Graduate	16 (24.6)	9 (19.2)
Total	65 (100.0)	47 (100.0)
*P* value	—	0.054
**Working for income**		
No	9 (12.2)	4 (8.2)
Yes	65 (87.8)	45 (91.8)
Total	74 (100.0)	49 (100.0)
*P* value	—	0.48
**Expressed financial concern**		
No	63 (85.1)	42 (85.7)
Yes	11 (14.9)	7 (14.3)
Total	74 (100.0)	49 (100.0)
*P* value	—	0.93
**Health insurance**		
No	0 (0.0)	3 (6.4)
Yes	72 (100.0)	44 (93.6)
Total	72 (100.0)	47 (100.0)
*P* value	—	0.030

FD, female donor; FND, female non-donor; MD, male donor; MND, male non-donor.

A higher percentage of FDs were non-related others (32/76, 42.1%) compared with MDs (13/49, 26.5%), but the overall distribution of relationships with recipients was not significantly different between these groups. Significant differences were observed when comparing FDs with MNDs (*P* < 0.001) and FNDs (*P* = 0.004), with a higher percentage of non-related others in the latter groups (749/1081 FNDs [69.3%], 317/556 MNDs [57.0%]).

There was no significant difference in the education level, employment status, and expression of financial concern between MDs and FDs. MDs were significantly more likely to not have health insurance (*P* = 0.030).

## Discussion

While there was considerable sex imbalance in living kidney donation, with 75 out of 125 female volunteers (60.8%) undergoing donor nephrectomy compared with 49 of 125 male volunteers (39.2%), we found that this began at the initial step of self-referral. In this study of 1861 PLDs at a single center, both sexes were equally likely to be declined as LDs because of medical and/or psychosocial contraindications and to be approved for donation. There was no difference between sexes in the risk of exclusion because of positive cross-matching. Furthermore, contrary to a similar study performed in 2005,^4^ both sexes were equally likely to follow through with donation once approved. However, with 1214 female and 647 male volunteers, females were 1.9 times as likely as males to volunteer themselves for evaluation, likely accounting for the observed sex difference in donation. Of note, female volunteers were more likely to be excluded because of ABOi (if the recipient did not wish to pursue paired exchange) and recipient-related factors. Given that these factors favor male donation, they do not explain female overrepresentation among LDs.

Many studies have documented sex differences across various aspects of ESKD management.^2–12^ On the recipient side, it has been established that females are less likely to be accepted for dialysis, placed on the KT waitlist, and receive KT.^4,6–9^ Simultaneously, female volunteers have consistently contributed to the LD pool at higher rates than male volunteers.^4–12^ Studies from several regions^6,8,10–12^ have found that female volunteers comprised 55%–65% of LDs. There are many conjectures for the existence of this phenomenon with sparse literature to support or negate them. For example, male volunteers are often hypothesized to be excluded more frequently because of medical contraindications, given that several chronic conditions are more prevalent among them.^6^ HLA sensitization through pregnancy in female recipients may prevent male spousal donation for some.^6^ Fear of income loss has been suggested as a barrier to male donation, under the assumption that males are the primary income earner.^6,8,9^ With few exceptions,^4,5^ studies of sex imbalance in LDKT have included only individuals who underwent donor nephrectomy, and not those who were excluded throughout the evaluation. This has significantly limited our ability to move beyond hypothesis and use a data-driven approach to facilitating male living donation. To our knowledge, following an extensive literature review, our cohort of 1861 PLDs constitutes the largest study of sex imbalance in the LD evaluation process and the first to incorporate data from the 2010s onward. Notably, in a prior study at our institution, comprising 506 PLDs between 2000 and 2004, both sexes were equally likely to be approved for donation, but male volunteers were less likely to proceed once approved.^4^ Crucially, this difference was only observed during the era of open nephrectomy (before November 2002),^4^ suggesting male volunteers were more likely to be deterred by the prospect of an open, but not laparoscopic, procedure. Our cohort, evaluated from 2009 onward, was entirely composed of individuals undergoing laparoscopic nephrectomy, which may explain why in our study, male volunteers were as likely as female volunteers to proceed with surgery.

Our findings suggest that female volunteers are more likely to express initial interest in LD evaluation compared with male volunteers. With the available data, we were not able to discern whether KT candidates also have a higher propensity to call upon female family members and friends, although this is certainly a possibility because studies have demonstrated the active role that many candidates play in recruiting their LDs.^14^ It is interesting to note that a higher percentage of female volunteers were in the non-related others category; this group consisted of non-spousal, non-biologically related individuals, such as friends, in-laws, and community members, perhaps suggesting that female volunteers are viewed as having a wider network of relational duty and commitment.^15^ We hypothesize that gender norms, particularly the view that females are more nurturing and altruistic, contribute to the observed imbalance in donation rates.^15–18^ However, qualitative research is needed to elucidate the nuances in the decision-making processes of PLDs and KT candidates: the former in their decision to step forward and the latter in whom they choose to engage. As importantly, our findings imply there may be an untapped pool of male individuals who are not currently being considered for donation, but would likely be eligible. Care teams should encourage KT candidates to discuss donation with a diverse group of individuals, including male individuals, to maximize chances of finding a LD. Community-level initiatives targeting male volunteers to raise awareness and enthusiasm for living donation may also prove effective. Additional work using a mixed-methods approach would certainly help refine these strategies and potentially expand access to LDKT, thereby alleviating the shortage of transplantable organs.

In comparing the characteristics of PLDs, FDs are significantly more White than non-donors with a difference of approximately 10%. It is also important to note that White and non-Hispanic individuals are overrepresented in all four comparison groups, relative to the racial distribution of Massachusetts.^19^ These findings support previously shown racial and ethnic disparities in living donation, highlighting the need for improved outreach to promote equitable access to care.^20^ In terms of the often-cited economic barriers to male donation, our data show no differences in FDs and MDs working for income and expressing financial concern related to donation; therefore, this likely did not play a role in the sex imbalance in our cohort.

Contrary to previous assumptions, both sexes in our cohort were equally likely to be declined because of medical contraindications. The top six comorbidities for both sexes accounted for approximately 80% of exclusions because of medical contraindications. These included BMI >32, uncontrolled hypertension, more than one lifetime occurrence of nephrolithiasis, concern for malignancy, and diabetes mellitus for both sexes. However, consistent with the sex predilection of both conditions,^6,21^ male volunteers were more often declined because of cardiovascular disease (as the fourth most frequent contraindication) and female volunteers, autoimmune disease (as the sixth most frequent contraindication). Notably, since the end of the study period, our institution has sought to expand access to LDKT by conducting further tests on individuals with history of nephrolithiasis and only excluding those with active stone formation or urine chemistries, suggesting increased risk of stone formation.

BMI >32 was, by far, the most frequently encountered medical contraindication to donation in both sexes, reflecting the growing epidemic of obesity in the United States. High-BMI donation poses an ethical and clinical concern because the long-term risk of hypertension and ESKD are slightly increased in obese individuals.^22^ However, the absolute risk of ESKD remains low at 0.7% in obese individuals, compared with 0.5% in non-obese individuals.^23^ In light of this, our institution has modified the exclusion criterion to BMI >35 with the goal of expanding the LD pool; this change did not go into effect until after the end of our study period. In addition, transplant centers may consider forming partnerships with independent weight loss and bariatric surgery clinics to help PLDs lose weight rather than upfront exclusion.^22,24^ Further work remains to be performed to explore the efficacy and ethics of these strategies.^21^

With two exceptions, there were no significant differences in the proportion by which male and female volunteers were being declined because of each specific contraindication. These were cardiovascular disease, which was more commonly seen in male volunteers (*P* = 0.035), and BMI >32, which was more commonly seen in female volunteers (*P* = 0.004). In Massachusetts, both conditions are less prevalent among females.^25–27^ Therefore, the latter finding suggests some discrepancy between the non-donors in our cohort and the general population. Although the rate of obesity is higher among females in Black and Hispanic populations,^25^ this is unlikely to explain the difference observed in our predominantly White sample. Granular socioeconomic variables may have provided useful context for interpreting this interesting finding.

It is crucial to consider that sex and gender are often invoked interchangeably.^6^ Sex refers to biological characteristics, while gender is a sociocultural construct, involving identities, behaviors, and relationships.^6^ While our medical record consistently documented sex, gender was only noted in a few instances (often when incongruent with sex). Therefore, we were restricted to using sex as a proxy for gender. After careful review, we surmised that our cohort included two transgender individuals, both men who had been assigned female at birth. Our discussion has proceeded under the imperfect assumption that gender coincided with sex for the other PLDs.

The limitations of our study otherwise include its retrospective design, introducing the possibility of misreported information and selection bias due to the availability of data within our institutional medical record and Organ Transplant Tracking Record database. It is possible, given the small number of individuals in certain categories (such as exclusion because of ABOi and recipient-related factors), that the significant difference between sexes may be due to type 1 error or may not accurately represent true population differences. In addition, there were 146 approved LDs of 1861 referrals, resulting in a conversion rate of 7.9%, which is slightly lower than that reported in the literature.^28^ This is likely due to strict selection criteria to ensure LDs were in excellent health. As noted, some of these have undergone changes since 2022 and are periodically adapted to reflect the evolving literature. Furthermore, as more granular socioeconomic variables only became available with formal psychosocial evaluation, we lacked these data for most of the non-donors, thus limiting our ability to compare these factors across the entire cohort. Finally, this study was performed at a single center; conducting a multicenter study spanning geographical regions is the next step in this investigation.

In conclusion, our institutional experience demonstrates that the overrepresentation of female volunteers among LDs for KT is due to the 1.9 times higher rate of female self-referral for evaluation. After this point, both sexes were equally likely to complete medical workup, have medical and/or psychosocial contraindications, be approved, and follow through with donation. This contradicts many assumptions that were previously made regarding the underlying reasons for sex imbalance in living donation.^9,10^ More importantly, it suggests that increased efforts to engage male volunteers has the potential to expand access to LDKT, which remains a much needed strategy to meet the demand for organs. As an avenue for further research, mixed-methods investigations are likely to be helpful in designing initiatives to increase male participation in living donation.

## Supplementary Material

## Data Availability

The data that support the findings of this study are openly available in the Lippincott Data Repository at http://links.lww.com/KN9/A680.
