# Supplementary material for: A Single-Center Retrospective Study to Identify Causes of Sex Differences in the Living Kidney Donor Evaluation Process
Source: Kidney360. 2024 Sep 16;5(12):1893–901. doi: 10.34067/KID.0000000581 (PMC11687984; doi:10.34067/KID.0000000581)
Supplement: Supplementary file 1 [file kidney360-5-1893-s001.pdf]

ASN American Society of Nephrology | JASN Journal of the American Society of Nephrology | KIDNEY360 Accessing Our World From Every Angle | CJASN Clinical Journal of the American Society of Nephrology

ASN Journal Disclosure Form

As per ASN journal policy, I have disclosed any financial relationships or commitments I have held in the past 36 months as included below. I have listed my Current Employer below to indicate there is a relationship requiring disclosure. If no relationship exists, my Current Employer is not listed.

S. Canizares Quisiguina reports the following:
Employer: Beth Israel Deaconess Medical Center

I understand that the information above will be published within the journal article, if accepted, and that failure to comply and/or to accurately and completely report the potential financial conflicts of interest could lead to the following: 1) Prior to publication, article rejection, or 2) Post-publication, sanctions ranging from, but not limited to, issuing a correction, reporting the inaccurate information to the authors' institution, banning authors from submitting work to ASN journals for varying lengths of time, and/or retraction of the published work.

Name: Stalin Isaias Canizares Quisiguina
Manuscript ID: K360-2024-000527R1
Manuscript Title: A Single-Center Retrospective Study to Identify Causes of Sex Differences in the Living Kidney Donor Evaluation Process
Date of Completion: September 4, 2024
Disclosure Updated Date: September 4, 2024

ASN American Society of Nephrology | JASN Journal of the American Society of Nephrology | KIDNEY360 Accessing Our World From Every Angle | CJASN Clinical Journal of the American Society of Nephrology

ASN Journal Disclosure Form

As per ASN journal policy, I have disclosed any financial relationships or commitments I have held in the past 36 months as included below. I have listed my Current Employer below to indicate there is a relationship requiring disclosure. If no relationship exists, my Current Employer is not listed.

B. Chopra has nothing to disclose.

I understand that the information above will be published within the journal article, if accepted, and that failure to comply and/or to accurately and completely report the potential financial conflicts of interest could lead to the following: 1) Prior to publication, article rejection, or 2) Post-publication, sanctions ranging from, but not limited to, issuing a correction, reporting the inaccurate information to the authors' institution, banning authors from submitting work to ASN journals for varying lengths of time, and/or retraction of the published work.

Name: Bhavna Chopra
Manuscript ID: K360-2024-000527R1
Manuscript Title: A Single-Center Retrospective Study to Identify Causes of Sex Differences in the Living Kidney Donor Evaluation Process
Date of Completion: August 21, 2024
Disclosure Updated Date: August 21, 2024

ASN American Society of Nephrology | JASN Journal of the American Society of Nephrology | KIDNEY360 Accessing Our World From Every Angle | CJASN Clinical Journal of the American Society of Nephrology

ASN Journal Disclosure Form

As per ASN journal policy, I have disclosed any financial relationships or commitments I have held in the past 36 months as included below. I have listed my Current Employer below to indicate there is a relationship requiring disclosure. If no relationship exists, my Current Employer is not listed.

R. Chumdermpadetsuk reports the following:
Employer: Beth Israel Deaconess Medical Center; and Ownership Interest: Spouse's ownership interests: ; ?? ?Microsoft; ?? ?Apple; ?? ?Tesla; ?? ?NVIDIA; ?? ?Meta; ?? ?Walmart; ?? ?Rocket Companies, Inc.

I understand that the information above will be published within the journal article, if accepted, and that failure to comply and/or to accurately and completely report the potential financial conflicts of interest could lead to the following: 1) Prior to publication, article rejection, or 2) Post-publication, sanctions ranging from, but not limited to, issuing a correction, reporting the inaccurate information to the authors' institution, banning authors from submitting work to ASN journals for varying lengths of time, and/or retraction of the published work.

Name: Ritah R. Chumdermpadetsuk
Manuscript ID: K360-2024-000527R1
Manuscript Title: A Single-Center Retrospective Study to Identify Causes of Sex Differences in the Living Kidney Donor Evaluation Process
Date of Completion: September 4, 2024
Disclosure Updated Date: May 20, 2024

ASN
American Society of Nephrology
JASN
Journal of the American Society of Nephrology
KIDNEY360
Accessing Our World From Every Angle
CJASN
Clinical Journal of the American Society of Nephrology

ASN Journal Disclosure Form

As per ASN journal policy, I have disclosed any financial relationships or commitments I have held in the past 36 months as included below. I have listed my Current Employer below to indicate there is a relationship requiring disclosure. If no relationship exists, my Current Employer is not listed.

D. Eckhoff has nothing to disclose.

I understand that the information above will be published within the journal article, if accepted, and that failure to comply and/or to accurately and completely report the potential financial conflicts of interest could lead to the following: 1) Prior to publication, article rejection, or 2) Post-publication, sanctions ranging from, but not limited to, issuing a correction, reporting the inaccurate information to the authors' institution, banning authors from submitting work to ASN journals for varying lengths of time, and/or retraction of the published work.

Name: Devin Eckhoff
Manuscript ID: K360-2024-000527R1
Manuscript Title: A Single-Center Retrospective Study to Identify Causes of Sex Differences in the Living Kidney Donor Evaluation Process,"
Date of Completion: August 21, 2024
Disclosure Updated Date: May 19, 2024

ASN American Society of Nephrology
JASN Journal of the American Society of Nephrology
KIDNEY360 Accessing Our World From Every Angle
CJASN Clinical Journal of the American Society of Nephrology

ASN Journal Disclosure Form

As per ASN journal policy, I have disclosed any financial relationships or commitments I have held in the past 36 months as included below. I have listed my Current Employer below to indicate there is a relationship requiring disclosure. If no relationship exists, my Current Employer is not listed.

D. Lee reports the following:
Employer: Beth Israel Deaconess; and Consultancy: Alpha Sights.

I understand that the information above will be published within the journal article, if accepted, and that failure to comply and/or to accurately and completely report the potential financial conflicts of interest could lead to the following: 1) Prior to publication, article rejection, or 2) Post-publication, sanctions ranging from, but not limited to, issuing a correction, reporting the inaccurate information to the authors' institution, banning authors from submitting work to ASN journals for varying lengths of time, and/or retraction of the published work.

Name: David Donghyung Lee
Manuscript ID: K360-2024-000527R1
Manuscript Title: A Single-Center Retrospective Study to Identify Causes of Sex Differences in the Living Kidney Donor Evaluation Process
Date of Completion: September 9, 2024
Disclosure Updated Date: September 7, 2024

ASN American Society of Nephrology · JASN Journal of the American Society of Nephrology · KIDNEY360 Accessing Our World From Every Angle · CJASN Clinical Journal of the American Society of Nephrology

ASN Journal Disclosure Form

As per ASN journal policy, I have disclosed any financial relationships or commitments I have held in the past 36 months as included below. I have listed my Current Employer below to indicate there is a relationship requiring disclosure. If no relationship exists, my Current Employer is not listed.

A. Montalvan reports the following:
Employer: Beth Israel Deaconess Medical Center

I understand that the information above will be published within the journal article, if accepted, and that failure to comply and/or to accurately and completely report the potential financial conflicts of interest could lead to the following: 1) Prior to publication, article rejection, or 2) Post-publication, sanctions ranging from, but not limited to, issuing a correction, reporting the inaccurate information to the authors' institution, banning authors from submitting work to ASN journals for varying lengths of time, and/or retraction of the published work.

Name: Adriana Montalvan
Manuscript ID: K360-2024-000527R1
Manuscript Title: A Single-Center Retrospective Study to Identify Causes of Sex Differences in the Living Kidney Donor Evaluation Process
Date of Completion: September 9, 2024
Disclosure Updated Date: September 9, 2024

ASN
American Society of Nephrology
JASN
Journal of the American Society of Nephrology
KIDNEY360
Accessing Our World From Every Angle
CJASN
Clinical Journal of the American Society of Nephrology

ASN Journal Disclosure Form

As per ASN journal policy, I have disclosed any financial relationships or commitments I have held in the past 36 months as included below. I have listed my Current Employer below to indicate there is a relationship requiring disclosure. If no relationship exists, my Current Employer is not listed.

M. Pavlakis reports the following:
Employer: Beth Israel Deaconess Medical Center; Vertex Pharmaceuticals; Merck; Transplant Genomics; CareDx; Consultancy: Merck, Vertex; and Research Funding: site PI on trial for Trugraf Genomics study TRULO; site PI for CareDx study.

I understand that the information above will be published within the journal article, if accepted, and that failure to comply and/or to accurately and completely report the potential financial conflicts of interest could lead to the following: 1) Prior to publication, article rejection, or 2) Post-publication, sanctions ranging from, but not limited to, issuing a correction, reporting the inaccurate information to the authors' institution, banning authors from submitting work to ASN journals for varying lengths of time, and/or retraction of the published work.

Name: Martha Pavlakis
Manuscript ID: K360-2024-000527R1
Manuscript Title: A Single-Center Retrospective Study to Identify Causes of Sex Differences in the Living Kidney Donor Evaluation Process
Date of Completion: August 21, 2024
Disclosure Updated Date: May 20, 2024
